# Purification, crystallization and preliminary X-ray diffraction analysis of the *Escherichia coli* common pilus chaperone EcpB

**DOI:** 10.1107/S2053230X15006354

**Published:** 2015-05-20

**Authors:** James A. Garnett, Mamou Diallo, Steve J. Matthews

**Affiliations:** a Department of Life Sciences, Imperial College London, South Kensington, London SW7 2AZ, England

**Keywords:** pili, Ecp, *Escherichia coli*

## Abstract

Pili are key cell-surface components that allow the attachment of bacteria to both biological and abiotic solid surfaces, whilst also mediating interactions between themselves. In *Escherichia coli*, the common pilus (Ecp) belongs to an alternative chaperone–usher (CU) pathway that plays a major role in both early biofilm formation and host-cell adhesion. The chaperone EcpB is involved in the biogenesis of the filament, which is composed of EcpA and EcpD. Initial attempts at crystallizing EcpB using natively purified protein from the bacterial periplasm were not successful; however, after the isolation of EcpB under denaturing conditions and subsequent refolding, crystals were obtained at pH 8.0 using the sitting-drop method of vapour diffusion. Diffraction data have been processed to 2.4 Å resolution. These crystals belonged to the trigonal space group *P*3_1_21 or *P*3_2_21, with unit-cell parameters *a* = *b* = 62.65, *c* = 121.14 Å and one monomer in the asymmetric unit. Molecular replacement was unsuccessful, but selenomethionine-substituted protein and heavy-atom derivatives are being prepared for phasing. The three-dimensional structure of EcpB will provide invaluable information on the subtle mechanistic differences in biogenesis between the alternative and classical CU pathways. Furthermore, this is the first time that this refolding strategy has been used to purify CU chaperones, and it could be implemented in similar systems where it has not been possible to obtain highly ordered crystals.

## Abstract 2

In *Escherichia coli*, the common pilus (Ecp) belongs to an alternative chaperone–usher pathway that plays a major role in both early biofilm formation and host-cell adhesion. Initial attempts at crystallizing the chaperone EcpB using natively purified protein from the bacterial periplasm were not successful; however, after the isolation of EcpB under denaturing conditions and subsequent refolding, crystals were obtained at pH 8.0 using the sitting-drop method of vapour diffusion. This is the first time that this refolding strategy has been used to purify CU chaperones.

## 1. Introduction   

Bacterial surfaces are decorated by sticky hair-like structures called fimbriae or pili that allow them to recognize abiotic surfaces, host receptors and also each other (Kline *et al.*, 2009 ▶; Proft & Baker, 2009 ▶). These interactions define the initial steps of colonization and the subsequent formation of biofilms: bacterial communities that are encased in a matrix that provides protection from external pressures such as antibacterial compounds and host clearance mechanisms (Croxen & Finlay, 2010 ▶). The majority of *Escherichia coli* are commensal strains that inhabit the bowels of animals and maintain a symbiotic relationship with their host; however, there are also a number of other strains that are highly pathogenic and can cause severe gastrointestinal and urinary-tract diseases (Croxen & Finlay, 2010 ▶). Although different strains of *E. coli* have developed specific pili to enable them to thrive in their niche environments, almost all produce a surface fibre called the *E. coli* common pilus (Ecp; Pouttu *et al.*, 2001 ▶; Rendón *et al.*, 2007 ▶; Garnett *et al.*, 2012 ▶). This structure is involved in key processes during sessile Entero­bacteriaceae lifecycles, where it mediates both host-cell adherence and early biofilm interbacterial interactions (Rendón *et al.*, 2007 ▶; Lehti *et al.*, 2010 ▶; Garnett *et al.*, 2012 ▶).

Biogenesis of Ecp is *via* an alternative chaperone–usher (CU) pathway (Waksman & Hultgren, 2009 ▶) and all genes necessary for this can be found on a single operon composed of *ecpR*, *ecpA*, *ecpB*, *ecpC*, *ecpD* and *ecpE* (Pouttu *et al.*, 2001 ▶; Garnett *et al.*, 2012 ▶). EcpR is a transcriptional regulator, while EcpC is an usher pore responsible for pilus assembly and secretion, and EcpA and EcpD are both components of the pilus. The majority of the Ecp shaft is composed of the 17.9 kDa EcpA pilin subunit. We have previously solved the X-ray crystal structure of this major pilus component and have further shown how it promotes inter-Ecp biofilm interactions through the antiparallel winding of fibres about one another (Garnett *et al.*, 2012 ▶). EcpD is an adhesive-tip subunit that can recognize receptors on the surface of host cells. It is the largest pilin subunit of all known CU systems (57.7 kDa) and also has the unique ability to self-polymerize (Garnett *et al.*, 2012 ▶; Rossez *et al.*, 2014 ▶). Another intriguing feature of the Ecp operon is that it expresses two chaperones rather than the usual single chaperone, which share ∼30% sequence identity: EcpB (22.4 kDa) and EcpE (23.7 kDa).

CU pilin domains can be thought of as incomplete Ig-like domains with unstructured N-terminal extensions (Sauer *et al.*, 2002 ▶; Zavialov *et al.*, 2003 ▶). During polymerization and export at the outer membrane usher, the N-terminal extension of one pilin lines the hydrophobic groove of an adjacent subunit completing the Ig-like fold, a process that has been termed donor-strand exchange (DSE; Remaut *et al.*, 2006 ▶). The role of the chaperone during this process is (i) to protect the exposed hydrophobic groove of the pilin to prevent its degradation and/or self-polymerization in the periplasm, (ii) to target the pilin subunits to the outer membrane usher pore and (iii) to synchronize DSE during pilus assembly. Within fibres of Ecp, both EcpA and EcpD must bury a large conserved tryptophan residue within the core of the adjacent subunit during DSE (Garnett *et al.*, 2012 ▶). The current mechanism of DSE that has been proposed for other pili formed through the classical CU pathway, however, is not consistent with this observation. As such, a subtle variation of DSE must exist in this alternative CU pathway and, in turn, differences should be observable in the structure of the free chaperones. Here, we present a new strategy for purifying CU chaperones that provides highly pure yields and was essential to facilitate the production of ordered crystals of free EcpB. Furthermore, we describe our preliminary crystallographic analyses of EcpB and envisage that the elucidation of its structure will further unravel the anomalies in this alternative CU pathway.

## 2. Materials and methods   

### 2.1. Cloning and expression   

Full-length EcpB (residues 1–202), minus the native N-terminal periplasmic signal sequence, was amplified from the genomic DNA of uropathogenic *E. coli* (UPEC) strain CFT073 and cloned into the N-terminal His_6_-tagged vector pET-46 Ek/LIC. This was transformed into *E. coli* strain BL21 (DE3), which was grown at 37°C in LB medium. Expression was induced with 1 m*M* isopropyl β-d-1-thiogalactopyranoside (IPTG) when an OD_600 nm_ of 0.6 was reached and was followed by growth overnight at 18°C (native purification) or 37°C (refolding purification).

### 2.2. Protein purification and crystallization   

For native purification of EcpB, cells were harvested and then resuspended in 20 m*M* Tris–HCl pH 8, 200 m*M* NaCl, 5 m*M* MgCl_2_, 1 µg ml^−1^ DNase I, 5 µg ml^−1^ lysozyme followed by sonication and nickel-affinity chromatography. For denatured purification of EcpB, cells were harvested and then resuspended in 20 m*M* Tris–HCl pH 8, 200 m*M* NaCl, 8 *M* urea followed by sonication. Denatured EcpB was then isolated using nickel-affinity chromatography in the presence of 8 *M* urea. The eluted protein was diluted to 20 µ*M* in resuspension buffer with the addition of 10 m*M* β-mercapto­ethanol and was then dialyzed against 20 m*M* Tris–HCl pH 8, 200 m*M* NaCl, 1 *M* urea followed by 20 m*M* Tris–HCl pH 8, 200 m*M* NaCl. Both natively purified and refolded EcpB were finally gel-filtered using a Superdex 75 column (GE Healthcare) and concentrated to 10 mg ml^−1^. Conditions for crystallization were initially screened by the sitting-drop method of vapour diffusion at 293 K using sparse-matrix crystallization kits from Hampton Research, Emerald Bio and Molecular Dimensions in MRC 96-well optimization plates (Molecular Dimensions) with 100 nl protein solution and 100 nl reservoir solution using a Mosquito nanolitre high-throughput robot (TTP Labtech). Protein crystals could only be obtained for refolded EcpB from 15%(*v*/*v*) glycerol, 15%(*w*/*v*) PEG 5000 MME after one week and were then manually optimized using MRC MAXI 48-well optimization plates (Molecular Dimensions) with 2 µl protein solution and 2 µl reservoir solution.

### 2.3. X-ray data collection and processing   

Crystals were mounted in a cryoloop and immediately flash-cooled in liquid nitrogen. Diffraction data from a single native crystal were collected on beamline I04 at the Diamond Light Source (DLS), England. Data were processed with *XDS* (Kabsch, 2010 ▶) and scaled using *SCALA* (Evans, 2006 ▶) within the *xia*2 package (Winter, 2010 ▶). Data-collection statistics are given in Table 1 ▶. The content of the unit cell was analyzed using the Matthews coefficient (Matthews, 1968 ▶). Molecular replacement was performed using *AMoRe* (Navaza, 2001 ▶), *MOLREP* (Vagin & Teplyakov, 2010 ▶), *Phaser* (McCoy, 2007 ▶) and within *MR_Rosetta* (Terwilliger *et al.*, 2012 ▶). High-resolution data were used between 2.4 and 6.0 Å and search models were prepared manually using *CHAINSAW* (Stein, 2008 ▶) as intact structures, as polyalanine models and with or without loop truncations. Furthermore, ensembles of these models were also used during molecular replacement.

## 3. Results and discussion   

**Figure 1:**
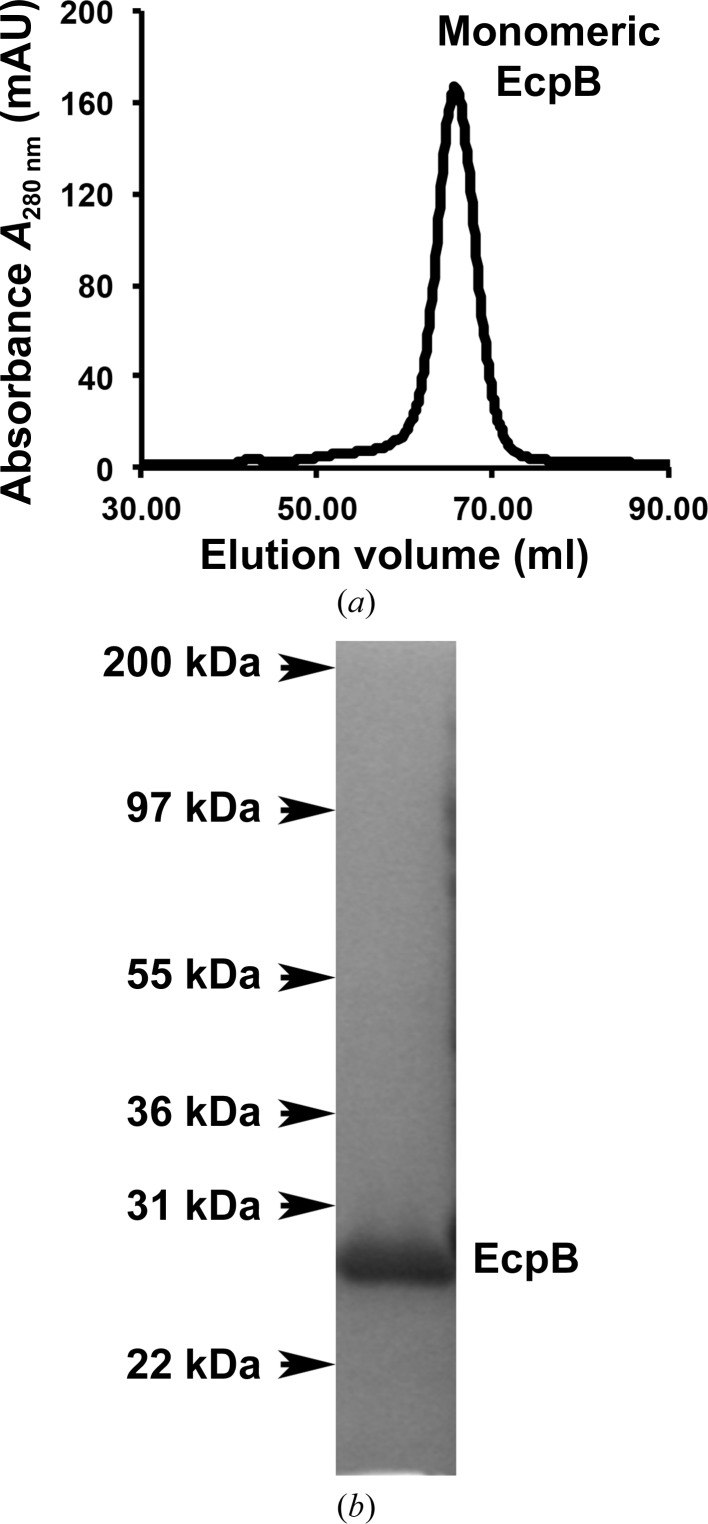
Purification of refolded EcpB. (*a*) Superdex 75 (GE Healthcare) gel-filtration profile of monomeric EcpB. (*b*) SDS–PAGE of EcpB after gel filtration.

**Figure 2:**
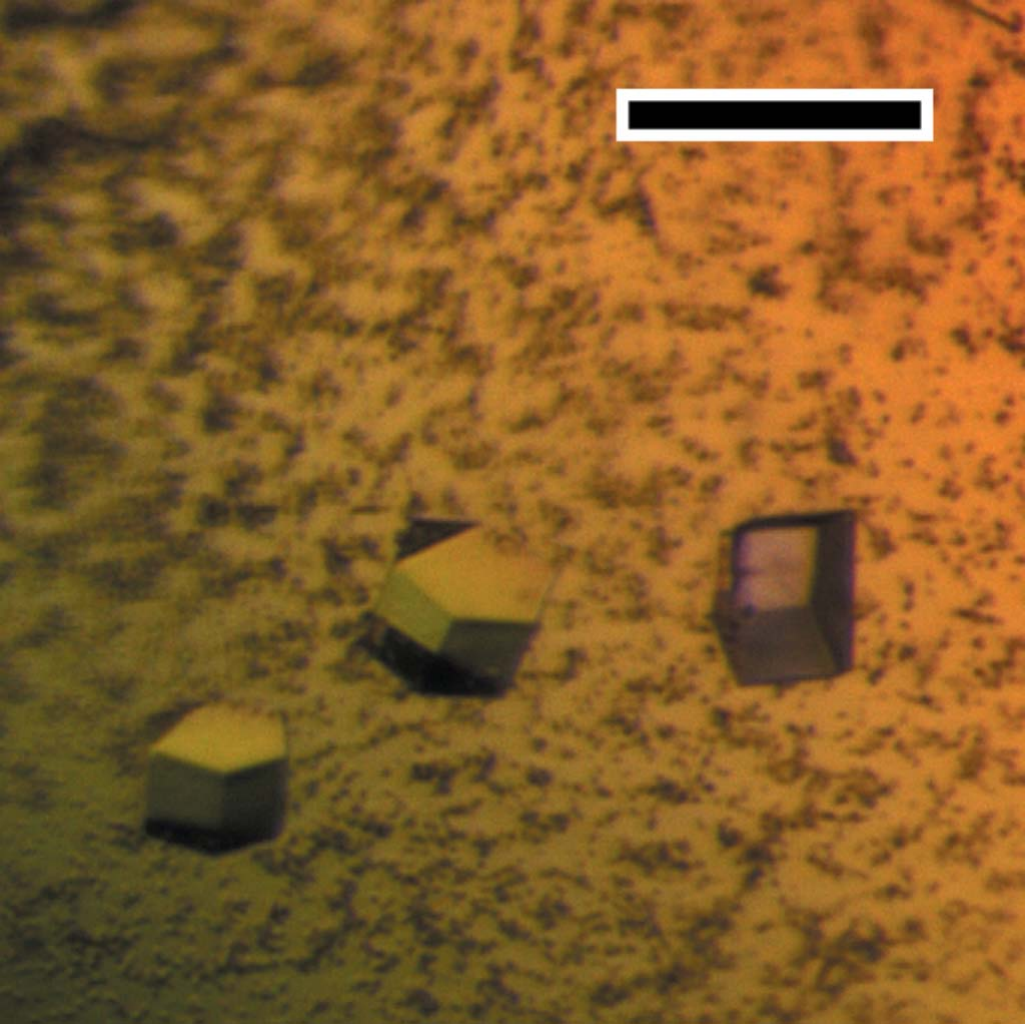
Representative native crystals of EcpB. The scale bar is 100 µm in length.

**Figure 3:**
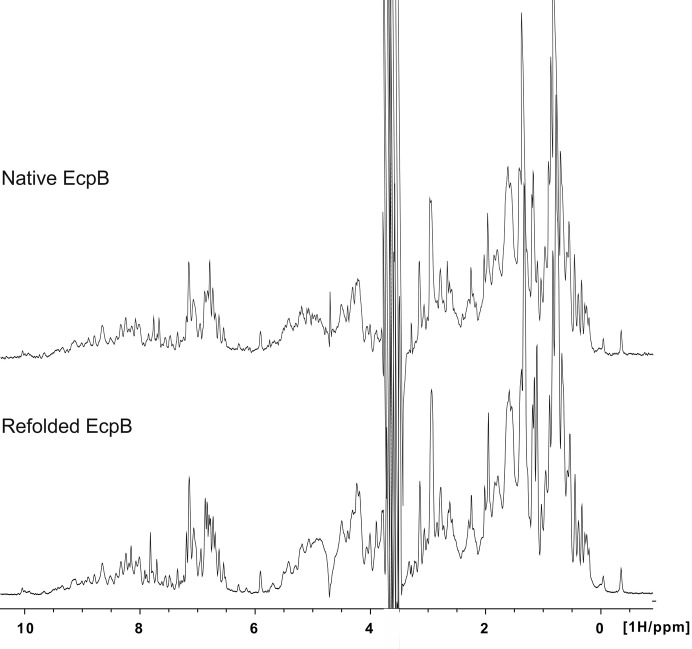
Comparison of natively purified and refolded EcpB using one-dimensional ^1^H NMR spectroscopy.

**Figure 4:**
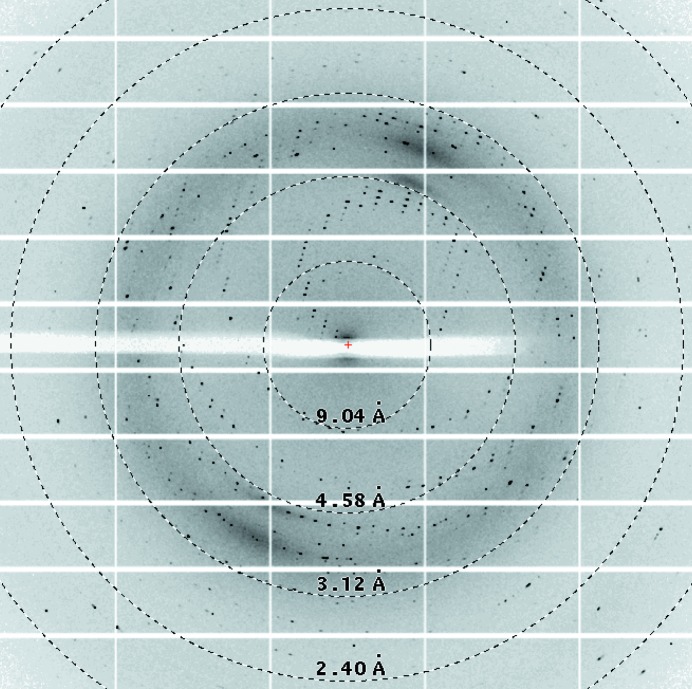
Diffraction image of an EcpB crystal. Resolution rings at 9.04, 4.58, 3.12 and 2.40 Å are annotated.

Crystal structures of chaperones from the CU pathway have always been obtained from native material purified directly from the periplasm (Waksman & Hultgren, 2009 ▶). Periplasmic production did not produce sufficient material for crystallization studies; therefore, EcpB was expressed in the cytoplasm and initially purified under native conditions. No suitable crystals were obtained from this sample despite exhaustive attempts. EcpB expression in a range of different conditions indicated that a significant amount of recombinant protein was also present as inclusion bodies; therefore, in a parallel approach we purified EcpB under denaturing conditions and subsequently refolded it with a view to increasing the yield and providing a cleaner preparation (Fig. 1 ▶). Crystals grew readily from this material to approximately 50 mm^3^ over the course of one week (Fig. 2 ▶). Comparison of natively purified and refolded EcpB using one-dimensional ^1^H NMR spectroscopy indicated that EcpB was fully folded in both preparations (Fig. 3 ▶), and as the protein spectra were indistinguishable we can conclude that the refolded sample is conformationally identical to native EcpB. It is therefore likely that the higher purity of the refolded sample is responsible for its improved crystallizability. Diffraction data were collected to ∼1.9 Å resolution (Fig. 4 ▶) and indexed in space groups *P*3_1_21 and *P*3_2_2; however, owing to a very high *R*_merge_ at full resolution the data were finally scaled at 2.4 Å resolution. Analysis of the crystal content indicated that there is a single molecule in the asymmetric unit with a Matthews coefficient of 2.75 Å Da^−1^ (Matthews, 1968 ▶) and a corresponding solvent content of 55%. This is supported by self-rotation function analysis and the presence of a single origin peak within a native Patterson function. Furthermore, the *L*-test suggests that twinning is not present (〈|*L*|〉 = 0.492). Data-collection and processing statistics are listed in Table 1 ▶.

Molecular replacement was attempted with *AMoRe* (Navaza, 2001 ▶), *MOLREP* (Vagin & Teplyakov, 2010 ▶), *Phaser* (McCoy, 2007 ▶) and within *MR_Rosetta* (Terwilliger *et al.*, 2012 ▶) using all known structures of chaperone–usher pathway chaperones as search models: CupB2 (PDB entry 3q48; Cai *et al.*, 2011 ▶), SafB (PDB entry 2co7; Remaut *et al.*, 2006 ▶), DraB (PDB entry 4djm; Z. Dauter, R. Piatek, M. Dauter & A. Brzuszkiewicz, unpublished work), FimC (PDB entry 1klf; Hung *et al.*, 2002 ▶), Caf1M (PDB entry 4ay0; Yu *et al.*, 2012 ▶), PapD (PDB entry 2xg5; Chorell *et al.*, 2010 ▶), CfaA (PDB entry 4ncd; Bao *et al.*, 2014 ▶), SfaE (PDB entry 1l4i; Knight *et al.*, 2002 ▶) and FaeE (PDB entry 3gfu; Van Molle *et al.*, 2009 ▶). Unfortunately, no solutions were found; however, the sequence identity between EcpB and these homologues is less than 20%. We are currently preparing selenomethionine-substituted protein and heavy-atom derivatives with a view to solving the phase problem using anomalous dispersion techniques. This example presents a new strategy for producing highly pure CU chaperones, particularly from this family, that could also be applicable to other systems where crystallization has not been successful.

## Figures and Tables

**Table 1:** Data-collection statistics Values in parentheses are for the highest resolution shell.

Space group	*P*3_1_21 or *P*3_2_21
Unit-cell parameters ()	*a* = *b* = 62.65, *c* = 121.14
Resolution ()	54.262.40 (2.462.40)
Wavelength ()	0.97949
Total reflections	216767 (15651)
Unique observations	11265 (805)
Completeness (%)	99.9 (99.7)
Multiplicity	19.2 (19.4)
*R*_merge_ †	0.057 (0.492)
*I*/(*I*)	44.4 (6.6)
Molecules per asymmetric unit‡	1
Solvent content (%)	55
Overall *B* factor from Wilson plot (^2^)	32.3

† *R*_merge_ = 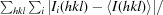 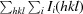, where *I*(*hkl*) is the mean intensity of the observations *I*_*i*_(*hkl*) of reflection *hkl*.

‡ Most probable value.
